# Photodetectors based on carbon nanotubes deposited by using a spray technique on semi-insulating gallium arsenide

**DOI:** 10.3762/bjnano.5.208

**Published:** 2014-11-05

**Authors:** Domenico Melisi, Maria Angela Nitti, Marco Valentini, Antonio Valentini, Teresa Ligonzo, Giuseppe De Pascali, Marianna Ambrico

**Affiliations:** 1 INFN – Sezione di Bari, Via Orabona 4, 70126, Italy; 2 Department of Physics, University of Bari “A. Moro”, Via Orabona 4, 70126, Italy; 3 CNR IMIP, UoS di BARI, via Orabona 4, Bari, 70126, Italy

**Keywords:** multi-wall carbon nanotubes, photodetectors, spray technique, UV–NIR

## Abstract

In this paper, a spray technique is used to perform low temperature deposition of multi-wall carbon nanotubes on semi-insulating gallium arsenide in order to obtain photodectors. A dispersion of nanotube powder in non-polar 1,2-dichloroethane is used as starting material. The morphological properties of the deposited films has been analysed by means of electron microscopy, in scanning and transmission mode. Detectors with different layouts have been prepared and current–voltage characteristics have been recorded in the dark and under irradiation with light in the range from ultraviolet to near infrared. The device spectral efficiency obtained from the electrical characterization is finally reported and an improvement of the photodetector behavior due to the nanotubes is presented and discussed.

## Introduction

Fast photoconductive detectors (PCD) are widely used for the characterization of sub-nanosecond pulses generated from infrared (IR) to ultraviolet (UV) light, X-ray and gamma-ray photons, as well as charged particles [1–3]. Applications of carbon nanotubes (CNTs) in this field have shown interesting results, in particular in new technologically advanced nanoelectronic devices [4–5]. Photodetectors based on films of CNTs (both bundle and carpet distribution) on silicon, have been previously analyzed in the visible and IR spectral regions [6–7]. Moreover the chemical, mechanical and electrical properties make CNTs also suitable to fabricate a wide range of radiation detectors for space applications, high energy physics and medical instrumentation [7–9].

The common technique obtain CNT films is chemical vapour deposition (CVD), but some deposition requirements, such as high temperatures of the substrates, the necessity of a catalyst and, as a consequence, the necessity of a barrier layer between the latter and the substrate, limit the applications of this technique [10–12]. In our previous work we have already shown the potential of a spray deposition technique for depositing CNTs on silicon, starting from a powder, at low temperatures, without catalyst and an intermediate layer [7]. By using this spray technique, CNT films on silicon-based photodetectors were prepared, achieving quantum efficiency (QE) values in the visible light range comparable with those obtained for similar detectors with CNTs deposited by CVD [7,13–15]. In this work results from a photodetector based on CNTs spray-deposited on semi-insulating gallium arsenide (SI GaAs) are reported.

In order to perform the morphological characterization of the resulting films, electron microscopy, in scanning (SEM) and transmission (TEM) modes, was used.

Current–voltage (I–V) characterizations under dark and illuminated conditions, from NIR to UV region, were performed with two different device configurations. The resulting QE and the photocurrent spectral measurements are reported and discussed. Finally, the effect of the nanotubes on the charge generation and collection in the detector is analyzed.

## Experimental

**Figure 1:**
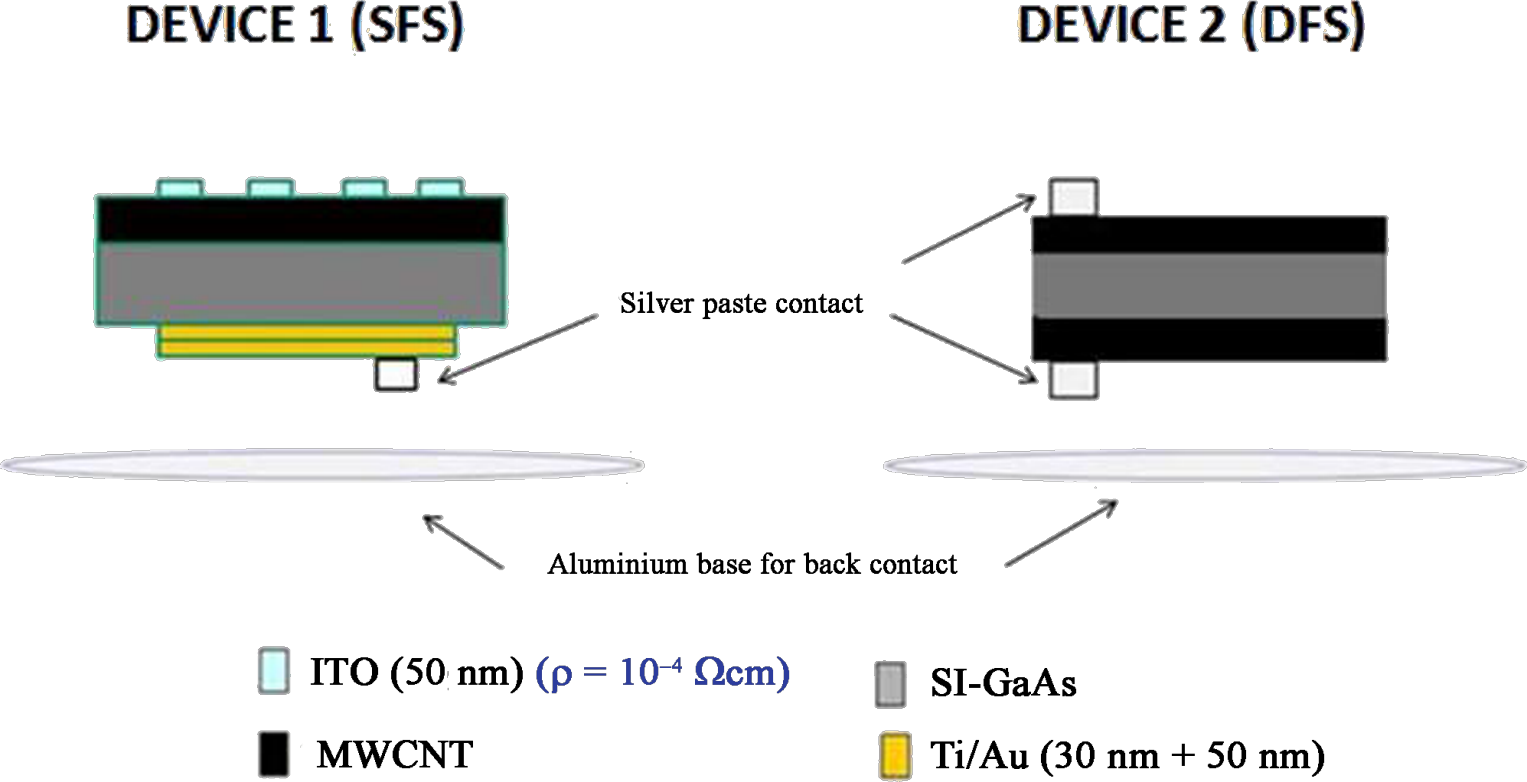
Schematic image of the two device layouts used: single face sample (SFS) and double face sample (DFS).

The multi-wall CNT (MWCNT) powder, with a purity degree greater than 95 wt %, was provided by COMETOX. The nanotubes are 5–15 μm long, and the diameters are between 10 and 30 nm. The procedure for the preparation of the solution is reported elsewhere [7]. The only difference is that an ultrasonic atomizer NS60K50-Sonaer 60 kHz system has been used in place of an airbrush, in order to obtain a better film uniformity. Due to the low deposition temperature (60 °C), custom-made transparent Mylar masks were used to better confine the CNT deposition area. SI GaAs substrates (350 ± 25 μm, resistivity 5.4·10^7^–9.1·10^7^ Ω·cm, Hall mobility 5692–6025 cm^2^·V^−1^·s^−1^, carrier concentration 1.2·10^7^–2.1·10^7^ cm^−3^), produced by Wafer Technology LTD, were used for the fabrication of the photodetectors. In order to obtain the final devices, the substrates were first degreased in acetone and methanol, after that they were etched for 30 s in a fresh solution of H_2_SO_4_/H_2_O_2_/H_2_O (4:1:1), then rinsed in methanol and double-distilled water and finally dried with nitrogen. Two different layouts were used for the realization of the photodetectors (Figure 1). The first configuration, named single face sample (SFS), has a CNT layer sprayed on one face and a titanium/gold layer (30/50 nm), deposited by ion beam sputtering (IBS) [16], on the other face. The second one, named double face sample (DFS), consists of a CNT layer sprayed on both sides. The SFS configuration with the presence of a Schottky contact (Ti/Au layer) on the GaAs substrate [16] was chosen as the final device, while the DFS has been realized and analyzed only to study the electrical characteristics of CNTs on the gallium arsenide. After the spray process, in the SFS device configuration, an interdigitated 50 nm thick indium tin oxide (ITO) film was deposited from an ITO target on CNTs by means of IBS. An ITO/GaAs/Ti/Au device was also prepared as control sample.

Transmission electron microscopy (FEI Tecnai G^2^ Spirit, 120 kV energy) was used to preliminarily evaluate the degree of dispersion of the CNTs in the spray solution performing few spray on TEM grids. Scanning electron microscopy (Zeiss-Sigma microscope with in-lens detector, 20 μm aperture, 10 kV energy) was used to obtain informations about the thickness of the CNT film and about the uniformity of its distribution on the GaAs substrate.

In order to perform the electrical characterization, all the samples were mounted gluing the back side on an aluminium disk by using a silver paste, leaving the front side covered with CNTs for the light exposure. The voltage supply was connected to the top ITO contact whereas the sample was grounded at the rear through the aluminium support.

For the calculation of QE, a typical configuration for spectral photocurrent measurements with a light source, wavelength selector and reference diode has been used. Specifically, a Thorlabs OSL1 white light source with optical fiber and a focusing lens were used to obtain a light spot on the photodetector while single wavelengths (400, 500, 600 and 700 nm) in the visible light range were selected by means of a filter set. For the UV characterization in the spectral range of 150–210 nm the measurements were performed under vacuum by using a McPherson 30 W Deuterium lamp coupled with a monochromator (Mc Pherson TM/Div. of S.I. Corp. mod. 234/302) as spectral light source. In this configuration the response of an UV-calibrated NIST diode (International Radiation Detectors, serial no. 97-527) was used for the QE calculation. An Agilent source/monitor unit was used to record the current–voltage characteristics.

To obtain more detailed information of the photodetector in the range of vis–NIR, photocurrent spectra of the SFS and of the ITO/GaAs/Ti/Au control sample were also performed in air, in the range of 350–1050 nm at various DC voltages, collected by means of a Stanford Research 850 lock-in amplifier equipped with a current preamplifier. Monochromatic, chopped (at a frequency of 13Hz) light impinging on the ITO/CNTs side of the samples was obtained by a tungsten halogen lamp coupled with an Acton Reasearch Spectra Pro 300i monochromator. The photocurrent response as a function of the light intensity at given DC voltages and wavelengths were obtained by inserting neutral filters of various optical density on the light path towards the sample. A calibrated THOR Labs photodiode (Thorlabs PM100D with a silicon photodiode S120VC) was used to normalize the sample photocurrent to the incident light intensity for all the measurements in the vis–NIR region.

## Results and Discussion

**Figure 2:**
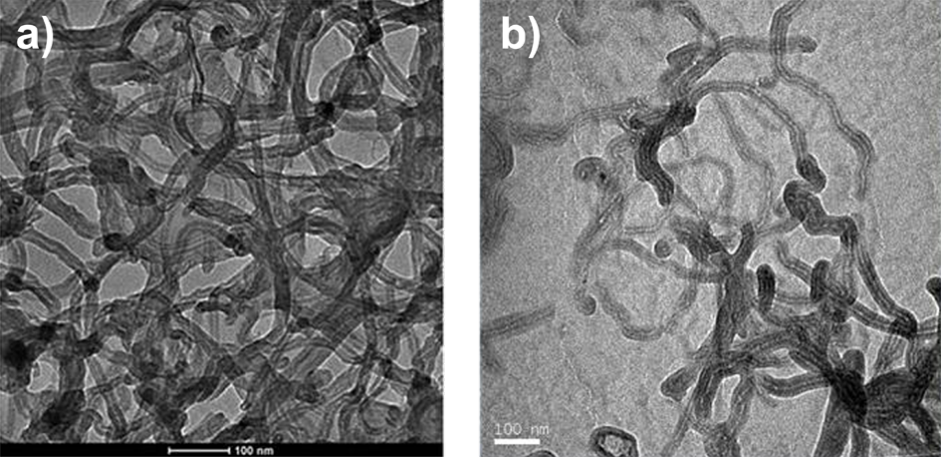
TEM micrographs of CNTs dispersion spray obtained by means of: the ultrasonic atomizer a); the airbrush b). Scale bars: 100 nm.

**Figure 3:**
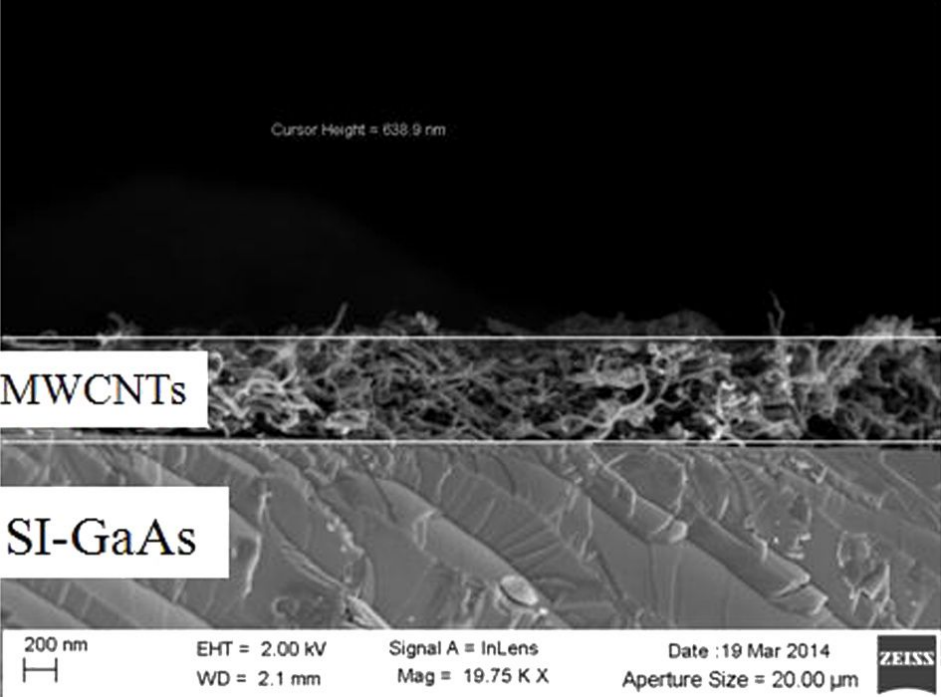
SEM image of the MWCNT film on a semi-insulating gallium arsenide substrate.

TEM images acquired at 120 kV of the spray dispersions obtained with both the atomizer (Figure 2a) and the airbrush (Figure 2b), show a more uniform distribution of the layer deposited with the atomizer uniform than that of the layer deposited with the airbrush. The uniformity of a thicker layer deposited on GaAs is also confirmed by the SEM image reported in Figure 3.

**Figure 4:**
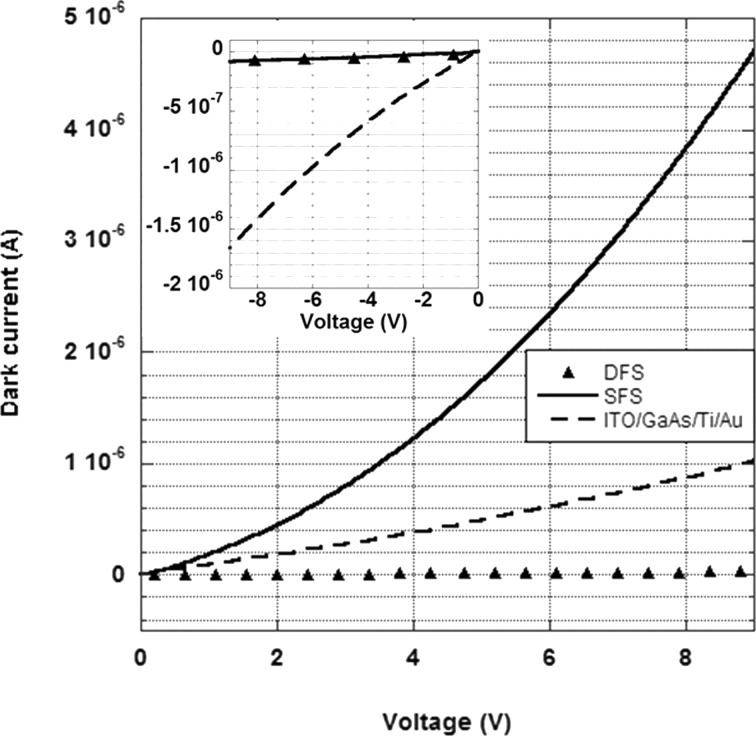
Dark-current–voltage characteristics for the SFS, DFS and ITO/GaAs/Ti/Au photodetector configurations. The inset reports the negative part of the dark-current–voltage characteristics.

In Figure 4 the dark-current–voltage (I–V) characteristics for the SFS, DFS and ITO/GaAs/Ti/Au photodetector configurations are reported. The inset reports the negative part of the I–V, showing the same trend for SFS and DFS samples. From the positive part a different behaviour of the SFS and DSF, due to the rectifying effect of Ti with respect to the ohmic effect of CNTs is observed. Good ohmic behaviour is demonstrated for the ITO/CNT/GaAs top contact of the SFS device. In fact, by comparing the rectifying and non-rectifying behaviour, respectively, of the SFS and of the ITO/GaAs/Ti/Au it can be deduced that the poor ohmic behaviour of the ITO/GaAs contact acts mostly as a counter barrier with respect to the GaAs/Ti/Au contact (Figure 4). In fact, for a positive applied voltage, the ITO/GaAs equivalent resistance increases, thus limiting the forward bias voltage drop at the GaAs/Ti/Au Schottky contact with subsequent reduction of the forward current. Then evidently the insertion of the CNT layer improves the ohmic behaviour with respect to the ITO/GaAs interface and consequently the diode performance.

The net photocurrents for negative bias of the devices in the vis–UV spectral region have been used to obtain the photoconductive absolute QE by means of the following formula:


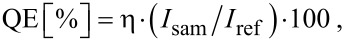


where *I*_sam_ [A] is the sample current, *I*_ref_ [A] is the calibrated photodiode current, and η is the internal quantum efficiency of the calibrated photodiode.

**Figure 5:**
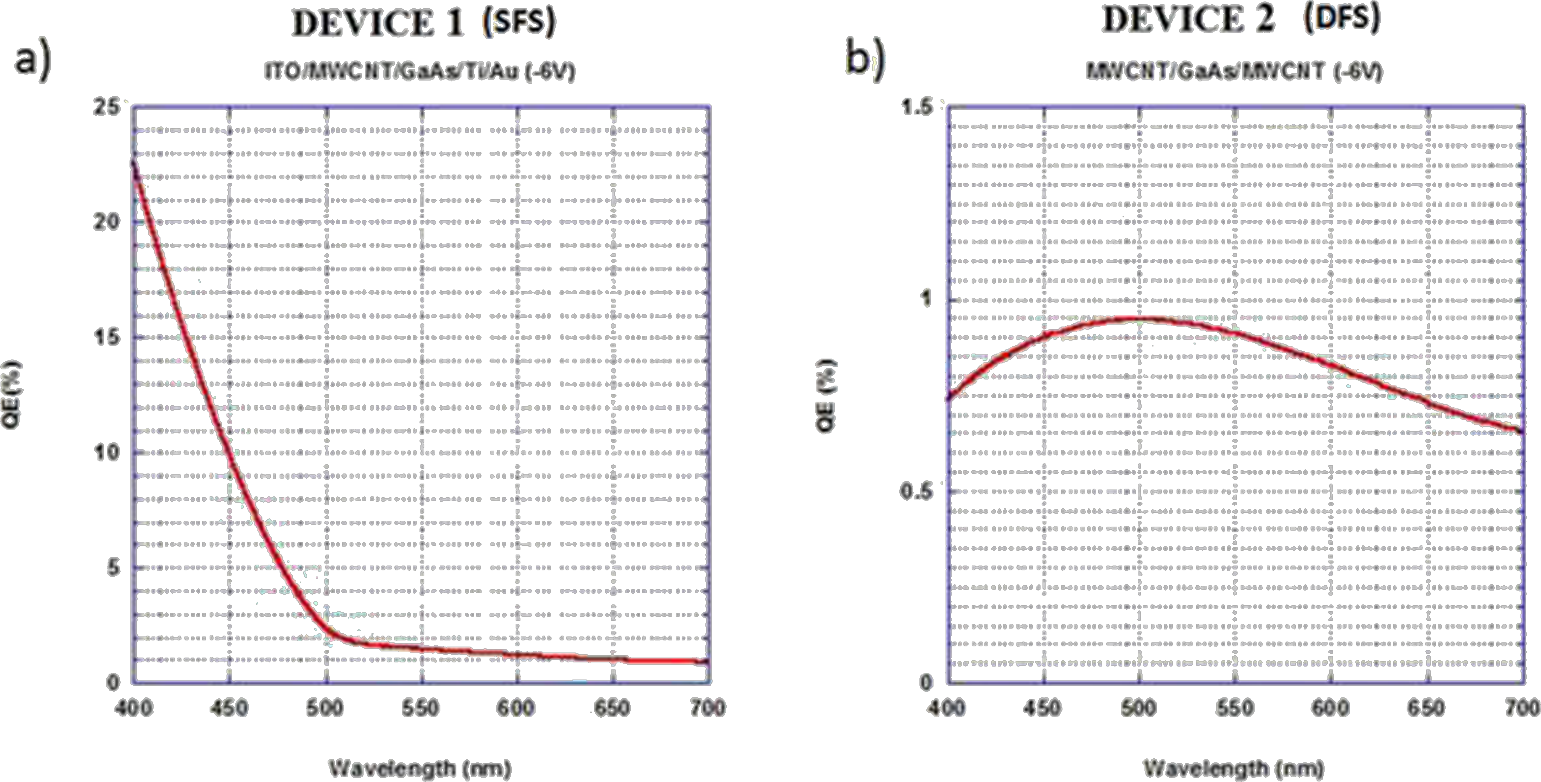
Absolute quantum efficiency trend in the visible light range, calculated at a bias voltage of −6 V for the devices SFS a) and DFS b).

**Figure 6:**
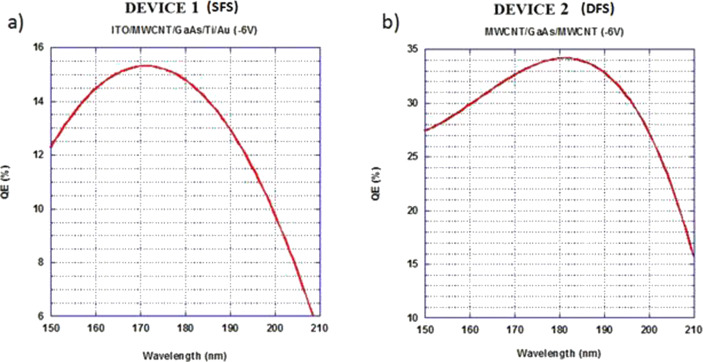
Absolute quantum efficiency trend in the UV range, calculated at a bias voltage of −6 V for the devices SFS a) and DFS b).

The resulting calculation trends, at a bias voltage of −6 V, are reported for SFS and DFS in Figure 5 and Figure 6 for the visible and the UV range, respectively. The higher QE of SFS with respect to DFS at lower wavelengths in the visible light range (Figure 5) can be attributed to the contribution to the photo-generated charges from the near-band-gap light absorption of ITO [17]. Furthermore, the interdigitated ITO contact of the SFS covers half of the active surface of the device, and its ultraviolet absorption gives rise to the halving of the QE values in this region (Figure 6).

**Figure 7:**
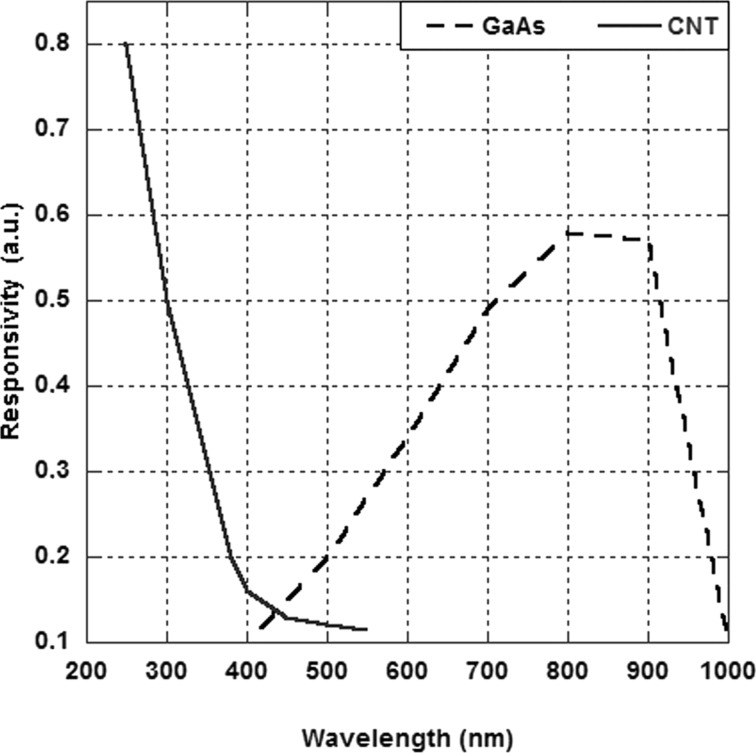
Responsivity trend of GaAs and CNTs based photodetectors.

To better understand the detector QE results in the range UV–vis that were analyzed so far, in Figure 7 [7] the expected responsivity of a gallium arsenide photodetector and, for comparison, a photodetector based on CNTs are reported. It is clear from the figure that the response in the UV of the CNTs/GaAs detector is due to the absorption of the CNTs in this region, to which, in the visible, the contribution of gallium arsenide is added.

**Figure 8:**
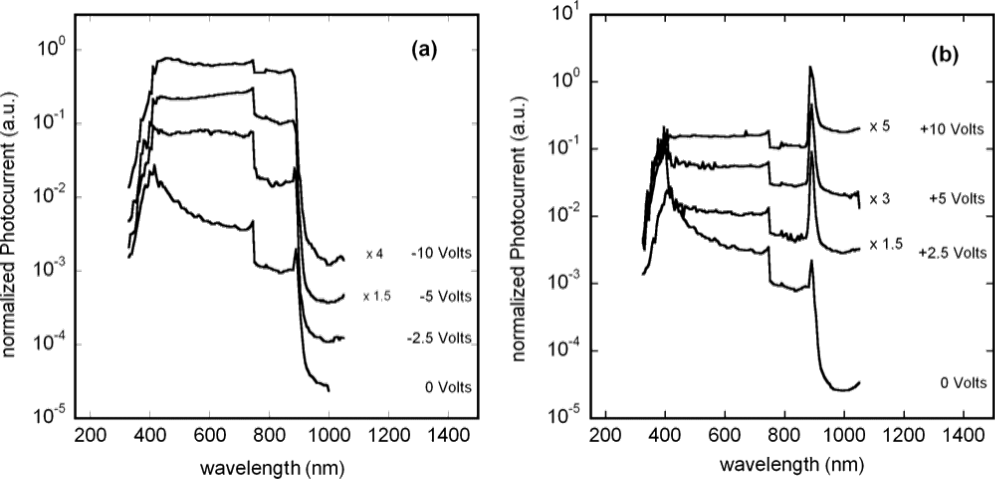
Normalized photocurrent spectra measured at: (a) negative voltages, (b) positive voltages applied to the ITO top contact with respect to the bottom Ti/Au contact. For the sake of clarity, some spectra were shifted up in the plots multiplying them by a factor, as indicated in the figure. The step shown in all the spectra at 750 nm is due to the change of the gratings of the monochromator.

**Figure 9:**
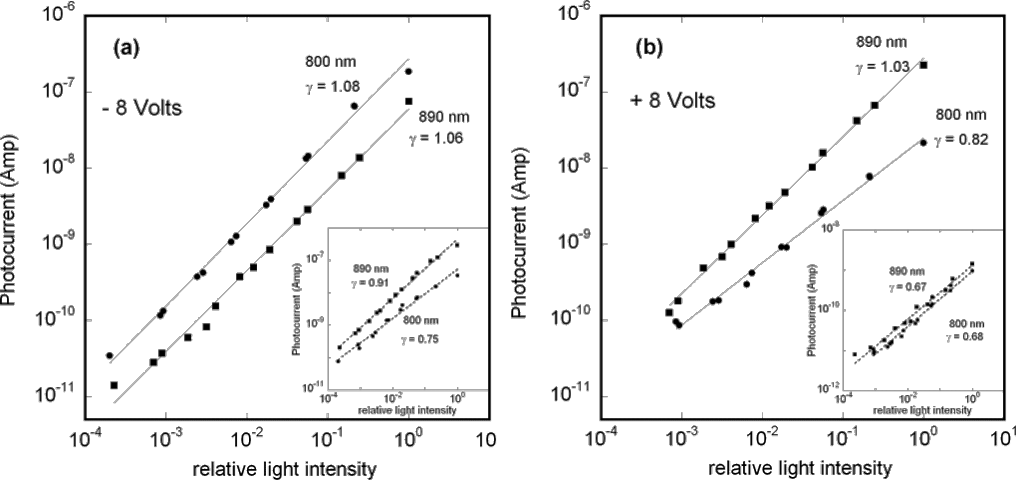
Photocurrent as a function of the relative monochromatic light intensity at λ = 800 and 890 nm for (a) −8 V and (b) +8 V applied to the top device contact. The best power-law fits are shown along with the resulting exponent γ. The full light intensity was approximately 10^−5^ W/cm^2^. In the insets (a) and (b) the results obtained for a similar device without CNTs (ITO/GaAs/Ti/Au) under the same experimental conditions are shown for comparison.

In Figure 8 the normalized photocurrent spectra, measured at various voltages, in the 350–1050 nm region are reported. In spite of the evident voltage-dependence of the spectra, all of them exhibit a steep increase in the IR range, between 900 and 1000 nm. Assuming the photocurrent directly proportional to the GaAs absorption coefficient and direct gap transitions, an absorption edge of 1.38 eV has been obtained for all the spectra. This value is slightly lower than the energy gap of GaAs at room temperature (1.42 eV) [18]. As mentioned above, the spectral response of the device is voltage-dependent and particularly the photocurrent increases linearly with the voltage in the NIR region, although at different rates for the various wavelengths: The closer to the GaAs absorbtion edge (1.38 eV), the more the photocurrent increases with voltage. This characteristic, with respect to what was observed in analogous photocurrent spectra of the ITO/GaAs/Ti/Au device (not shown), is more pronounced so that at the maximum investigated voltages (+10 and −10 Volts) the SFS spectra become nearly flat (Figure 8a and Figure 8b) in the whole vis–NIR range except for the pronounced peak at the absorption edge. This peculiarity and especially the fact that, for positive voltages only, the photocurrent onset at 900–1000 nm is greatly enhanced with respect to the whole investigated spectral range, is still under investigation. As a further observation it must be pointed out that, with respect to the ITO/GaAs/Ti/Au comparison sample, at the maximum of the absorption edge (890 nm), the photocurrent exhibits a linear dependence on the incident light intensity (i.e., 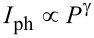 with γ ≈ 1) over many order of magnitude, as shown in Figure 9a and Figure 9b, for both negative and positive voltages. At 800 nm, only for positive voltage, the dependence turns out to be sublinear (γ = 0.82), as shown in Figure 9b. This result has to be compared with γ ranging between 0.67 and 0.91, obtained for the ITO/GaAs/Ti/Au in the same experimental conditions. To have a linear dependence of the photocurrent on the light intensity is an important achievement in a photodetector. Nevertheless in such devices (either p-i-n or Schottky diodes) the value of the exponent γ depends not only on the intrinsic properties of the photoactive material (traps and recombination centres distribution) but also on the properties of the contacts. Generally, the photocurrent [19] can be expressed as *I*_ph_ = *e*·*G*·*F*, where *F* is the number of electron–hole pairs photogenerated per unit time and *G* is the photoconductive gain representing the number of carriers passing between electrodes in the unit time for each absorbed photon in the unit time. In fact, depending on the type of the contacts (blocking, ohmic or injecting), while the photogenerated charge is swept away by the electric field, the photoconductor volume can be replenished with other carriers (secondary photocurrent). If both mobile photogenerated electrons and holes are not replenished at the electrodes [19], e.g., in a reverse-biased photodiode, *G* is at most unity and it occurs when the one photogenerated electron–hole pair per photon is swept out before recombining via a recombination centre. In this case γ = 1 as for the reverse-biased SFS diode (Figure 9a). Conversely, for a direct-biased SFS diode (Figure 9b) the ITO/CNT ohmic contact supplies extra majority carriers to the GaAs volume (possibly the forward-biased Ti/Au Schottky contact might also inject minority carriers). In this case *G* can be nominally larger than unity but the detector is less sensitive to the light intensity, i.e., γ ≤ 1 [20]. This occurs also for the ITO/GaAs/Ti/Au device as shown in the insets of Figure 9a inset and Figure 9b. As mentioned above for the dark-current–voltage characteristics, the ITO/GaAs contacts constitutes a counter barrier with respect to the GaAs/Ti/Au Schottky contact, so for a reverse bias of the latter the former is forward biased and viceversa, with secondary photocurrent generation [19–20] and γ < 1.

## Conclusion

In this work we have shown the capability to produce photodetectors based on MWCNTs/semi-insulating GaAs by using a spray technique to deposit the nanotube film. Two different configurations have been analysed. The first with a Ti/Au back contact (SFS) and a CNT film on the other face. The second with CNTs on both sides (DFS). Furthermore an ITO/GaAs/Ti/Au device was prepared to better understand some experimental results obtained for the SFS.

The *I*–*V* measurements under illumination evidence, in both configurations, the contribution of the responsivity of the CNTs in the UV as photoactive layer to the detector performance. Furthernore, in the vis–NIR spectral range photocurrent appears to be more field-dependent in the device with CNTs, so that at maximum applied voltages photocurrent spectra of the SFS (differently from the ITO/GaAs/Ti/Au) gets nearly flat. This aspect is still under investigation.

The comparison of the dark-current–voltage characteristics of the ITO/CNT/GaAs/Ti/Au (SFS) and of the ITO/GaAs/Ti/Au demonstrates the good ohmic behaviour of the ITO/CNT/GaAs contact with respect to the bare ITO/GaAs one. This peculiarity improves the rectifying properties of the SFS device and, as a further consequence, its linear photocurrent-dependence behaviour, especially in reverse-bias mode.
